# Biomaterial Embedding Process for Ceramic–Polymer Microfluidic Sensors

**DOI:** 10.3390/s20061745

**Published:** 2020-03-21

**Authors:** Witold Nawrot, Karol Malecha

**Affiliations:** Department of Microsystems, Wrocław University of Science and Technology, Wybrzeże Stanisława Wyspiańskiego 27, 50-370 Wrocław, Poland; witold.nawrot@pwr.edu.pl

**Keywords:** low-temperature cofired ceramic (LTCC), polydimethylsiloxane (PDMS), surface modification, selective plasma, biosensor, microfluidics

## Abstract

One of the major issues in microfluidic biosensors is biolayer deposition. Typical manufacturing processes, such as firing of ceramics and anodic bonding of silicon and glass, involve exposure to high temperatures, which any biomaterial is very vulnerable to. Therefore, current methods are based on deposition from liquid, for example, chemical bath deposition (CBD) and electrodeposition (ED). However, such approaches are not suitable for many biomaterials. This problem was partially resolved by introduction of ceramic–polymer bonding using plasma treatment. This method introduces an approximately 15-min-long window for biomodification between plasma activation and sealing the system with a polymer cap. Unfortunately, some biochemical processes are rather slow, and this time is not sufficient for the proper attachment of a biomaterial to the surface. Therefore, a novel method, based on plasma activation after biomodification, is introduced. Crucially, the discharge occurs selectively; otherwise, it would etch the biomaterial. Difficulties in manufacturing ceramic biosensors could be overcome by selective surface modification using plasma treatment and bonding to polymer. The area of plasma modification was investigated through contact-angle measurements and Fourier-transform infrared (FTIR) analyses. A sample structure was manufactured in order to prove the concept. The results show that the method is viable.

## 1. Introduction

Modern analytical procedures used in chemical, biological, or biochemical studies require specialized laboratory equipment. As a result, they are complex and time-consuming, while the number of places where the analyses can be performed is significantly reduced. The solution to these disadvantages is being widely developed under the concepts of micro total analysis system (μTAS) or lab-on-chip (LoC). These devices allow performing various analyses of biological or chemical agents in real time and automatically. Decrease of the analyte volume to micro- or nanoliters can significantly reduce the cost and time of the analysis and is beneficial to the environment due to the reduction of waste. The first concept of such miniature microfluidic systems for chemical analysis appeared in the early 1990s. Originally, silicon and glass were used as a construction material for μTAS and LoC fabrication. In the literature, there are examples of such structures, including a microreactor for polymerase chain reaction (PCR) [1] or an integrated miniature gas chromatograph [2]. Micromechanical and microfluidic structures with characteristic dimensions of up to tens of micrometers were developed in silicon using technological methods typical for semiconductor technology, such as wet anisotropic etching, anodic bonding, or diffusion. However, the significant cost of materials and technological equipment forced researchers to look for new technologies that would be adequate for the fabrication of such devices. Currently, these analytical chips are fabricated mainly in polymers and silicones, for instance, polycarbonates, polyimides, and polysiloxanes. However, the main drawback in the fabrication of microfluidic systems using these materials is the difficulty in assembly with electronic and optoelectronic components. On the other hand, ceramic microsystems excel in this area. Moreover, they offer several advantages for sensor manufacturing, most important of which are chemical inertness, temperature stability, fine structuration, and advanced electronic integration. A series of microfluidic optical sensors were already shown using this technology. A variety of channels and chambers can be integrated into ceramic structures in a width range from ca. 100 µm to several mm. The incorporation of optical structures is more challenging. Nevertheless, several solutions were shown, mostly utilizing glass windows with sapphire [3] or soda-lime [4]. However, the most promising solution is the method for bonding transparent polydimethylsiloxane (PDMS) to low-temperature cofired ceramic (LTCC) material. A hybrid PDMS/LTCC microsystem takes advantage of both materials. On one hand, fine fluidic structures can be made in a transparent polymer lid using laser micromachining, hot embossing, or soft lithography. On the other hand, a ceramic substrate with integrated conductors, passives, heaters, Micro Electro-Mechanical Systems (MEMS), actuators, optoelectronics components, and fluidic structures can be fabricated using the LTCC technology.

The low-temperature cofired ceramics technology is based on building spatial structures layer by layer from flexible sheets of an unfired ceramic–glass composite (GreenTape). Passive electronic components can be applied on each layer, such as conductive paths, resistors, capacitors, or coils, as well as active layers, such as sensors. The tape can also be structured and stacked together, to form complex spatial structures. The low-temperature cofired ceramics (LTCC) technology was proven to be eligible for microsystem [5,6] and biosensor development [7,8]. However, one of the key steps in the LTCC manufacturing process is sintering at temperatures of ca. 800–900 °C. As a result, the incorporation of temperature-sensitive (bio)chemical agents (e.g., catalytic bed, enzyme carrier, or polymeric receptor layer) is impossible, as they would decompose. Currently, this issue is being overcome by deposition of the biolayer from liquid after the thermal treatment [9], using methods such as chemical bath deposition (CBD) or electrodeposition (ED). Nevertheless, this approach is not suitable for many biomaterials and, therefore, a new technique of enclosing biostructures needs to be developed.

Polydimethylsiloxane is a transparent, macromolecular organosilicon polymer. Its core chain is composed of –Si–O– bounds, and it occurs in a linear or a cyclic form. Its properties are well known; it is considered as a neutral compound, approved for contact with all human tissues and use in foods and pharmacy. It is, thus, a very appropriate material for biosensor manufacturing. Moreover, it has several desirable features: high transmittance in a wide optical range, reflectivity coefficient similar to glass, good mechanical properties, and high chemical resistivity. The relatively easy formation, combined with inexpensive manufacturing materials and equipment, resulted in the high popularity of PDMS microfluidic devices. The surface of polydimethylsiloxane is dominated by nonpolar methyl groups, making it a hydrophobic material. Therefore, water-based debris is less likely to deposit on its surface, which improves both clarity and sanitation, especially in dye-based microfluidic sensors. On the other hand, this property causes difficulties in the deposition of biomaterials and bonding with other materials.

**Figure 1:**
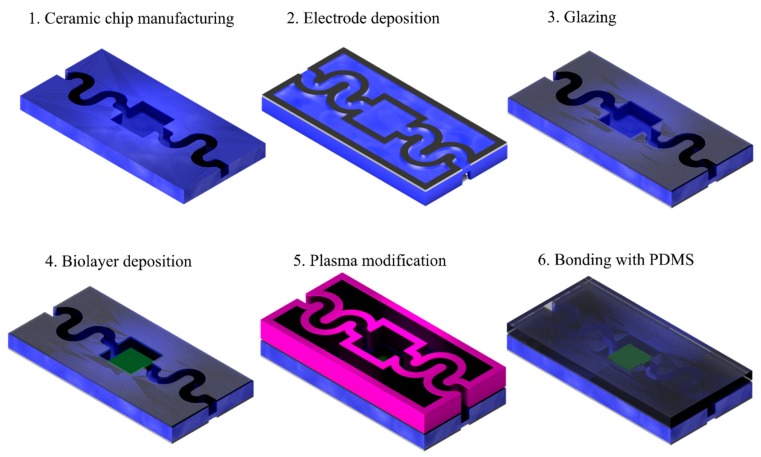
A process diagram of selective low-temperature cofired ceramic (LTCC)/polydimethylsiloxane (PDMS) bonding with biomaterial integration. (**1**) A ceramic microfluidic chip is manufactured; (**2**) a lower dielectric barrier discharge (DBD) electrode is deposited on the bottom of the chip; (**3**) the contact surface is glazed; (**4**) the biolayer is deposited inside the microfluidic chamber; (**5**) the contact surface is selectively modified using plasma, which appears only above the bottom electrode; (**6**) the plasma-modified PDMS is brought into contact with the modified surface and starts to bond with the glazed surface.

LTCC is a well-known technique for microfluidic sensors fabrication. However, the high temperature of the LTCC process results in serious limitation of this technology (e.g., it precludes fabricating biosensor with immobilized enzymes or polymeric receptor layers). Moreover, in many cases, the chemical analysis is based on observations of the phenomenon occurring within the microsystem (mixing, fluorescence, color changing, transport of particles suspended in flowing medium), and non-transparent LTCC materials preclude this type of study. To solve this problem, our group developed a method of LTCC with transparent PDMS bonding using DBD (dielectric barrier discharge) argon or argon/oxygen plasma [10]. The transparent cover allows for analysis of fluid flow and mixing inside the channels and chambers, which is an invaluable tool in microsystem diagnostics. Additionally, the polydimethylsiloxane cover can also contain fluidic systems. It can be molded from any form with very fine details, which is significantly less expensive than manufacturing silicon sensors. According to our previous research, the modified surfaces of LTCC and PDMS are stable only for 5–10 min. Therefore, the time for different operations, such as deposition of the sensing layer or introducing a catalytic bed to the microchannel fabricated in the LTCC substrate, is insufficient. The enzyme immobilization process can be very time-consuming, as it can last even a few hours. Therefore, a modified bonding procedure had to be developed. The problem was overcome by developing the process of selective modification of the LTCC surface. In this process, the biolayers can be immobilized in a specific area of the LTCC substrate (e.g., microchannel) before plasma modification. After immobilization, the LTCC substrate can be sealed with a PDMS cover plate using the DBD plasma process. The proper design of an electrode deposited on the bottom of the LTCC substrate allows for selective plasma occurrence. As a result, the modification takes place only in these regions of the LTCC where the biolayer was not immobilized. Afterward, both materials can be bonded together. Therefore, the general aim of this work is to introduce a novel method based on selective plasma activation after the biomodification step. The process is shown in Figure 1.

The method of bonding LTCC and PDMS was previously described [10,11]. In this process, the surface is modified through plasma treatment. This method was successfully applied in several sensors and microfluidic devices, allowing for optical measurements. Those devices can be highly complex, yet easy to manufacture. The first example was a device for absorbance measurement [12], which was composed of a microfluidic mixer and two optical windows. The core with channels was manufactured in ceramics, and the bottom and the top were made of polymer for transparency. The structure was surrounded by two light-emitting diodes and two light sensors for inexpensive, yet precise, absorbance measurements at two wavelengths. The bond between LTCC and PDMS was very strong, and the fluid did not leak or spill. It was also long-lasting, whereby it did not disintegrate over a control period of three years, and it kept its original optical properties. Another example is a microfluidic sensor [13], which allowed for fluorescence measurements in a ceramic microsystem.

The earlier-described LTCC/PDMS manufacturing method is, therefore, very promising for biosensing. However, in order to introduce new biomaterials using this technology, a method for bonding after the sensing layer deposition step was needed. It was crucial for the discharge to occur selectively; otherwise, the biomaterial would decompose. Furthermore, the process had to be carried out at room temperature, so that no thermal decomposition of material could occur. This was the reason for developing this method. It is very important to emphasize that this is the first method for non-invasive embedding of open ceramic channels after biomodification, at ambient temperature. As mentioned before, the area in which the plasma occurs is defined by the shape of an electrode on the bottom of the structure. It can surround the biomaterial, in order to provide a hermetic seal, while keeping the discharge away from the biolayer region. One of the major goals of this work is to assess if the free radicals generated by the plasma can migrate outside of the area defined by the electrode and interact with the biomaterial. The uniformity of modification plays a vital role for further reliability and, therefore, was also analyzed. Moreover, the optimization of the manufacturing process is also described, along with applications in sensor technology.

## 2. Materials and Methods

### 2.1. Materials

The polydimethylsiloxane layer was prepared from a Dow Sylgard^®^ 184 system. A base, containing >60 wt.% PDMS [14], was mixed in a 10:1 ratio with a cross-linker. Then, the mixture was degassed, transferred into molds, and left for cross-linking for 48 h at room temperature. The cross-linking process can be accelerated to 10 min at 150 °C; however, the slower process ensures the best fidelity to the mold.

The low-temperature cofired ceramics structures, in a variety of shapes, were developed using a DuPont GreenTape 951 system. Each layer, 254 μm thick, was cut using a Nd:YAG laser, operating at 355 nm (LPKF ProtoLaser U). Then, layers were laminated at a pressure of 20 MPa at a temperature of 70°C, using an isostatic press. Electrodes for selective discharge were screen-printed using 325-mesh screens on a VS 1520A (Aurel) machine. The electrodes were made of either gold 5742 (DuPont) or platinum 9141 (DuPont) thick film pastes. Structures were fired using a DP 951 suggested profile, with a peak temperature of 850 °C. The glaze layer SG-683K (Heraeus) was screen-printed using a 200-mesh screen in a post-firing step. It requires a lower firing temperature (ca. 500 °C); otherwise, it becomes very reactive and mixes with conductive and substrate layers during cofiring, which degrades the quality of the device. Afterward, the structure was fired with a peak temperature of 700 °C, in order to ensure a flat and even surface of the glaze.

### 2.2. Modification Procedures

The basis for this method is the modification of surface properties through plasma treatment. Free radicals present in plasma cause the methyl groups from polydimethylsiloxane to tear out and, thus, become radicals themselves. They are replaced by polar hydroxyl groups (OH) and oxygen radicals (O•), which introduce hydrophilicity to the surface [15,16]. Unfortunately, this state is short-lived and lasts only for several minutes.

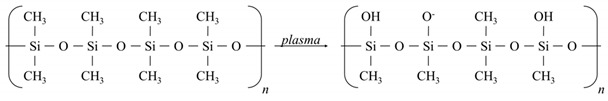
.(1)


After such modification, PDMS can be bonded to LTCC, using oxygen [10] or argon [11] plasma. Before the process, a glaze layer is screen-printed in order to reduce surface roughness and to introduce oxygen functional groups. Their presence is a result of water adsorption to glass at elevated temperature after firing. The glaze layer is also treated with plasma in order to remove residue and decompose OH–O bonds. As a result, more hydroxyl groups, which then turn into silanol groups, are present on the surface [17]. The bonding occurs after contact of the PDMS and the LTCC surfaces through dehydration. Strong and permanent covalent Si–O–Si bonds are, thus, formed as follows:

≡ Si–OH + OH–Si ≡ → ≡ Si–O–Si ≡ + H_2_O. (2)

**Figure 2:**
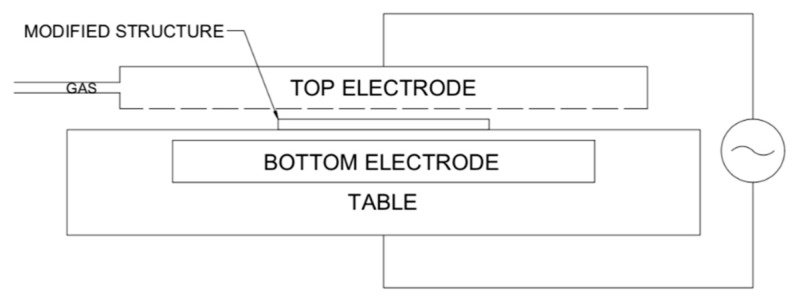
A schematic of the plasma modification reactor based on dielectric barrier discharge appearing between the bottom and top electrode under applied voltage. A modified structure is placed in between, with a working gas flowing through the top electrode.

Typical plasma modification for LTCC–PDMS bonding is carried out using dielectric barrier discharge (DBD) in a reactor, as shown in Figure 2.

**Figure 3:**
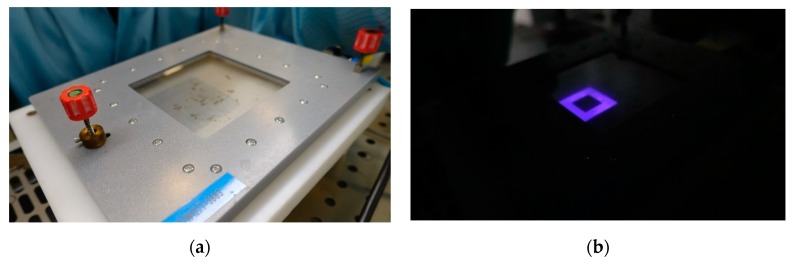
A view of the reactor for selective plasma modification: (**a**) before and (**b**) after plasma ignition. The window allows for observation of the discharge fidelity.

It consists of a table with an integrated bottom electrode, where a modified structure lays, and a top electrode, made of steel mesh, which allows for the flow of the working gas (i.e., oxygen or argon). Electrodes are connected to a power supply, operating at a voltage of U = 10 kV and a frequency f = 100 kHz. In this set-up, the plasma appears on the whole surface of the structure which, as mentioned earlier, would cause the biolayer to disintegrate. Therefore, a novel set-up was developed, in which the bottom electrode is not embedded in the table. Instead, a conductive layer is screen-printed on the bottom of a modified structure and connected to the power supply, acting as a bottom electrode. As mentioned before, it is crucial that the electrode area avoids the biolayer (Figure 1). The set-up is shown in Figure 3.

Of course, the thick film layer can take any shape, and the discharge appears only between two electrodes. Thus, the biological material is not directly affected by plasma. However, the question still remains whether generated free radicals are transported to the biolayer and interact with it. In order to answer this question, contact-angle measurements and Fourier-transform infrared (FTIR) analysis were carried out.

### 2.3. Characterization Procedures

The occurrence of surface modification was analyzed by contact-angle measurements inside and outside of the modification area, using a PGX goniometer (Fibro System AB). Measurements were carried out in a clean room environment at a relative humidity of 51% and at a temperature of 20.7 °C. Test structures were rinsed in deionized water, and then a series of at least 30 drops were analyzed, before and after modification, both inside and outside the modification area. Measurements were carried out firstly using water and then diiodomethane (Sigma-Aldrich), in order to calculate surface free energy (SFE) along with its dispersive and polar components. The SFE values and its components were calculated on the basis of Wu’s method [18,19], as the harmonic method is considered more accurate than that of Owens and Wendt [20]. Results of the contact-angle measurements are summarized in Table 1.

Modified structures were also analyzed by Fourier-transform infrared spectroscopy (FTIR) with an attenuated total reflection (ATR) crystal, using a Vertex 70v spectrophotometer (Bruker Optic GmbH).

## 3. Results

**Figure 4:**
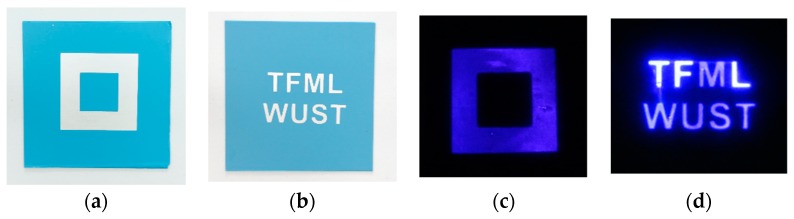
The fidelity of the selective dielectric barrier discharge to the shape of bottom electrodes, deposited on developed structures: (**a**) rectangular electrode, (**b**) letter-shaped electrode, (**c**) rectangular discharge, (**d**) letter-shaped discharge.

The very first task in the research was to obtain a selective discharge. In order to achieve this, a novel set-up was designed and manufactured, as described in the previous chapter. The fidelity of the discharge to the shape of the electrode was very good, as shown in Figure 4.

**Figure 5:**
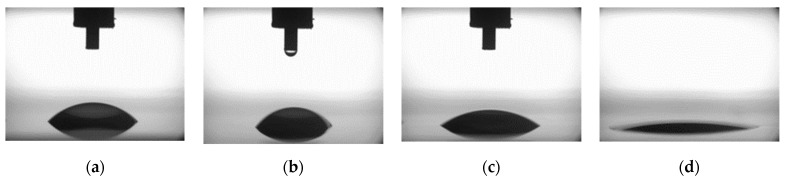
The sample contact angles measured outside (**a**,**b**) and inside (**c**,**d**) of the discharge area before (**a**,**c**) and after (**b**,**d**) the modification, using water droplets. A change in the appearance is visible only in the case of the measurement after the modification, inside the discharge area.

As mentioned before, one of the major aims of this work was to assess whether free radicals present in plasma induced by the selective dielectric barrier discharge can be transferred outside the discharge area and interact with surfaces. It is crucial for determining if the biomaterial surrounded by such discharge is safe from degradation. Fortunately, free radicals are highly reactive, which leads to rapid disappearance via reaction with themselves or with other substances [21]; therefore, they cannot travel far from their source, which is the plasma. This was confirmed by the results of contact-angle measurements, which clearly showed that modification occurred only inside of the discharge area, as presented in Figure 5. Taking into account that the wetting angle changes due to interaction with free radicals, it is highly probable that they do not migrate significantly and would not interact with biological material. The decomposition of biomaterial under plasma treatment is also a result of interaction with free radicals. Therefore, the assessment of their transport outside of the plasma region by measuring wetting angles is a viable basis to determine if the biolayer is safe. If the radicals traveled outside of the plasma area, they would interact with any surface, regardless of whether it is a biolayer or glass/ceramic. Results of contact-angle measurements are given in Table 2.

The measurements outside the modification area were carried out as close as possible to this area in order to detect even the shortest transport of the radicals. Based on these results, surface free energy was calculated as given in Table 3.

**Figure 6:**
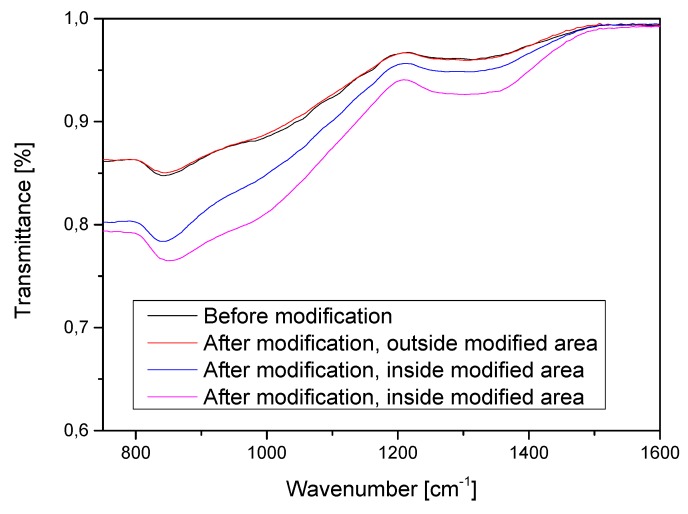
The Fourier-transform infrared (FTIR) spectra recorded before and after the plasma modification inside and outside of modified area. The change, which represents a reaction between free radicals and the surface, can be observed only after the modification, inside of the discharge area.

The results of contact-angle measurements were also in line with Fourier-transform infrared spectroscopy (FTIR) measurements. Three measurements were carried out inside the discharge area: one before and two after it occurred. One additional measurement was carried out outside of the discharge area for reference. The spectra before the modification and outside of the discharge area are highly similar (Figure 6). The most significant difference in the spectra can be observed at ca. 800–1400 cm^−1^, which corresponds to the absorption band of Si–OH and other Si–O functionalities [22]. The blue and pink lines represent the same experiment, but the measurement was performed at two points inside the modified area in order to prove that the change in surface composition was not accidental. However, those measurements were carried out sequentially after plasma modification. Increased surface reactivity after such alteration is an unstable state, as the process windows lasts for about 15 min. Thus, the difference in intensity might be a result of recovery to the default state or a sign of non-uniform modification.

**Figure 7:**
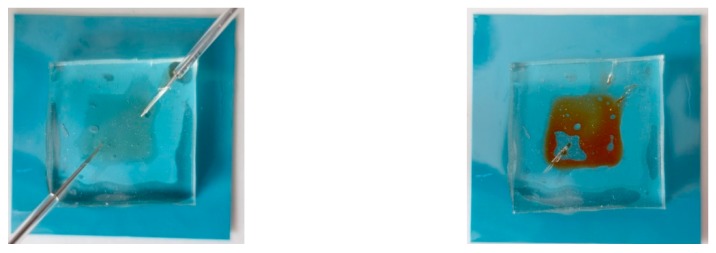
A view of the test microfluidic deice, before (**left**) and after (**right**) filling with methyl orange. An irregular bonding area can be observed, although the structure was hermetic.

Due to promising surface modification results, a sample microfluidic chamber was developed as a proof of concept. It allowed validation of hermeticity and comparison between the discharge and the bonding areas. Results show that the bonding was not uniform. This may be a result of insufficient power in the developed set-up or its uneven distribution, waviness of the substrate, or surface debris. The successful bonding of LTCC and PDMS was proven before; therefore, a careful optimization of the process should provide better quality. Nevertheless, the obtained bonding area was sufficient for the structure to be leakproof, as shown in Figure 7. More importantly, no bonding occurred outside of the modification area, which once again indicates the selectivity of the method. The shape was reasonably reproduced, while further optimization of the process should provide better results. Above all, this proves that the presented technology can bond PDMS to LTCC selectively around the biomaterial area, which would stay intact; therefore, it is eligible for biosensor manufacturing.

**Figure 8:**
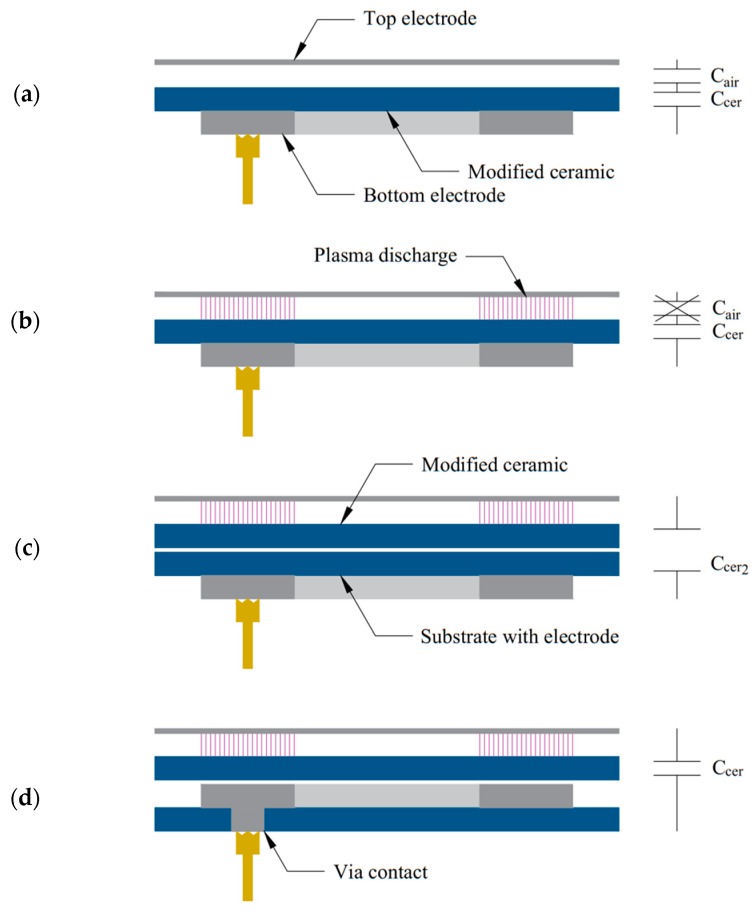
Selective dielectric barrier discharge (SDBD) set-ups for optimization of the electric field magnitude: (**a**) the bottom electrode deposited on the modified structure (capacitances before discharge occurrence); (**b**) air capacitance is broken during discharge; (**c**) for series manufacturing, the bottom electrode can be deposited on another substrate, although it increases capacitance during discharge, decreasing the magnitude of the electric field; (**d**) the bottom electrode can be deposited on top of the additional substrate to reduce additional capacitance, where contact is maintained through a via contact.

The optimization of plasma power distribution on the substrate surface can be carried out through minimization of capacitances in the set-up. In the process, the bottom electrode (which provides selectivity) can be printed either directly onto the modified structure (Figure 8a) or onto a separate substrate (Figure 8c). It was found that a typical FR4 laminate with copper metallization can also act as a bottom electrode, bringing down the cost of manufacturing. The second scenario is better suited for manufacturing a series of devices, as screen-printing of the bottom electrode is not needed on each device, decreasing the number of manufacturing steps. On the other hand, it introduced additional distance between electrodes, reducing the magnitude of the electric field, which can lead to inconsistent bonding, as shown before. However, the process can be optimized by adding a via contact through the electrode substrate and placing it upside down compared to the previous scenario (Figure 8d). In this way, the distance between electrodes was reduced to the thickness of the modified structure only, as the capacitance of air is irrelevant after the plasma occurs (Figure 8b). Therefore, the scenario presented in Figure 8d yields benefits of a small capacitance and a reduction in the number of manufacturing steps.

**Figure 9:**
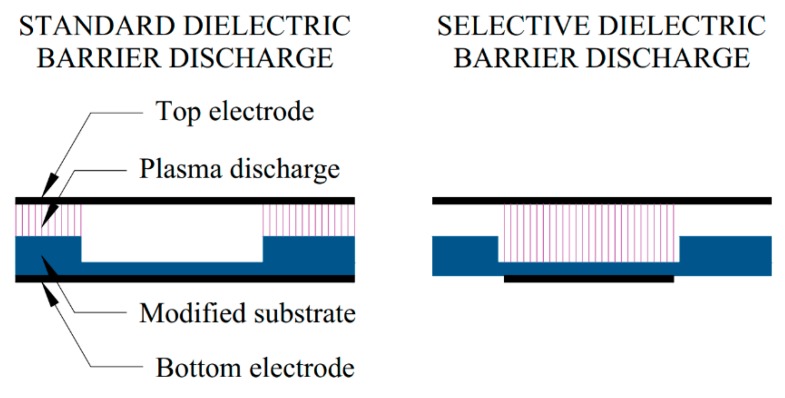
Application of the Selective Dielectric Barrier Discharge for bonding PDMS in cavities, which is difficult to obtain in a non-selective set-up.

Another application for described selective plasma is bonding in cavities. In a typical set-up, the discharge would occur at the shortest distance possible, i.e., at the top of the structure, as shown in Figure 9. By using the tailored electrode shape, it is possible to obtain the discharge in any desired area.

Placing PDMS in cavities introduces several benefits. It simplifies alignment, which is especially important if microfluidic channels are both in LTCC and PDMS parts. It screens undesired light, which is the key for optical sensors to work properly, especially for fluorescence measurements. Moreover, it shields the bond from tensile stresses, increasing robustness. On the other hand, building cavities requires more material. Furthermore, the outer layer of PDMS can act as a cushion for brittle ceramics or glass, especially in shock scenarios. Therefore, neither method is favorable in general, but rather dependent on the application.

**Figure 10:**
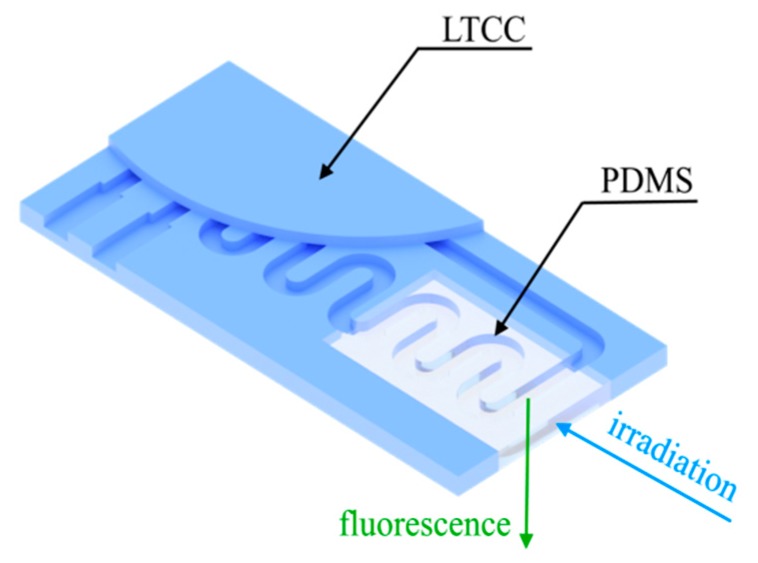
An example of PDMS implementation as both a waveguide and an optical window in a fluorescence sensor.

An exemplary sensor that can be manufactured using the described method is shown in Figure 10. PDMS can simultaneously act as both a waveguide and an optical window, being an ideal solution for fluorescence measurements, very common in biosensors. Moreover, the procedure described in this work allows for deposition of the biolayer on the LTCC substrate before bonding with PDMS.

## 4. Conclusions

A novel method for selective surface modification was described. It allows for selective bonding of ceramics or glass and silicone-based polymers. Therefore, embedding of temperature-sensitive structures, such as biological matter, is possible after deposition in open channels. Thorough analyses show that generated free radicals do not migrate out of the plasma region, avoiding biomaterial degradation. The described method allows for application of a wider range of biomaterials in LTCC/glass–PDMS technology, significantly increasing the potential of biosensor manufacturing. For example, urease, which is used for urea sensing, can now be embedded in an LTCC–PDMS microsystem using selective plasma bonding. This represents significant progress in this technology as such sensors are in high demand. The key benefits of the technology are the optical transparency of polymers such as polydimethylsiloxane (PDMS) and the extensive electronic packaging capabilities of ceramic microsystems such as in the low-temperature cofired ceramic technology (LTCC). Moreover, the introduced cavity bonding allows for new microsystem architectures. Furthermore, both PDMS and LTCC are proven microfluidic materials, well suited for contact with biomatter. As described earlier, LTCC was proven to be more than suitable for biosensor manufacturing. This new technology further extends its capabilities. Future work should utilize the described method.

## Figures and Tables

**Table 1:** Surface free energy values for liquids used for contact-angle measurements.

Test Fluid	Total ($\gamma_{L}^{}$) (mJ/m^2^)	Dispersive ($\gamma_{L}^{d}$) (mJ/m^2^)	Polar ($\gamma_{L}^{p}$) (mJ/m^2^)
Water	72.8	21.8	51
Diiodomethane	50.8	48.5	2.3

**Table 2:** Contact-angle measurement mean results.

	Water		Diiodomethane	
	Inside the Area	Outside the Area	Inside the Area	Outside the Area
Before modification	70.36 (σ = 5.65)	69.77 (σ = 5.09)	52.79 (σ = 1.47)	49.37 (σ = 1.34)
After modification	<10	70.75 (σ = 4.89)	27.62 (σ = 1.58)	47.12 (σ = 2.21)

**Table 3:** Surface free energy of the substrate.

	Total ($\gamma_{L}^{}$) (mJ/m^2^)	Dispersive ($\gamma_{L}^{d}$) (mJ/m^2^)	Polar ($\gamma_{L}^{p}$) (mJ/m^2^)
Before modification	49.3	35.4	13.9
After modification	82.4	45.5	36.8
